# Assessing changes in vascular permeability in a hamster model of viral hemorrhagic fever

**DOI:** 10.1186/1743-422X-7-240

**Published:** 2010-09-16

**Authors:** Brian B Gowen, Justin G Julander, Nyall R London, Min-Hui Wong, Deanna Larson, John D Morrey, Dean Y Li, Mike Bray

**Affiliations:** 1 Institute for Antiviral Research and Department of Animal, Dairy, and Veterinary Sciences, Utah State University, Logan, Utah, USA; 2 Program in Molecular Medicine, University of Utah, Salt Lake City, Utah, USA; 3 Integrated Research Facility, National Institute of Allergy and Infectious Diseases, National Institutes of Health, Fort Detrick, MD, USA

## Abstract

**Background:**

A number of RNA viruses cause viral hemorrhagic fever (VHF), in which proinflammatory mediators released from infected cells induce increased permeability of the endothelial lining of blood vessels, leading to loss of plasma volume, hypotension, multi-organ failure, shock and death. The optimal treatment of VHF should therefore include both the use of antiviral drugs to inhibit viral replication and measures to prevent or correct changes in vascular function. Although rodent models have been used to evaluate treatments for increased vascular permeability (VP) in bacterial sepsis, such studies have not been performed for VHF.

**Results:**

Here, we use an established model of Pichinde virus infection of hamsters to demonstrate how changes in VP can be detected by intravenous infusion of Evans blue dye (EBD), and compare those measurements to changes in hematocrit, serum albumin concentration and serum levels of proinflammatory mediators. We show that EBD injected into sick animals in the late stage of infection is rapidly sequestered in the viscera, while in healthy animals it remains within the plasma, causing the skin to turn a marked blue color. This test could be used in live animals to detect increased VP and to assess the ability of antiviral drugs and vasoactive compounds to prevent its onset. Finally, we describe a multiplexed assay to measure levels of serum factors during the course of Pichinde arenavirus infection and demonstrate that viremia and subsequent increase in white blood cell counts precede the elaboration of inflammatory mediators, which is followed by increased VP and death.

**Conclusions:**

This level of model characterization is essential to the evaluation of novel interventions designed to control the effects of virus-induced hypercytokinemia on host vascular function in VHF, which could lead to improved survival.

## Background

A number of RNA viruses in the filovirus, bunyavirus, arenavirus and flavivirus families cause a syndrome of fever, increased vascular permeability (VP), shock and hemorrhage in humans that is designated viral hemorrhagic fever (VHF). In most cases, alterations in vascular function is attributed to the release of cytokines and other permeability factors that destabilize the endothelial barrier, causing plasma volume loss that leads to hypovolemic shock, multi-organ failure, and death [1,2]. In the clinical setting, an increase in the hematocrit (HCT) is a marker for increased VP [3]. Because serum albumin is a highly abundant small molecular-weight protein, it may be particularly prone to diffusing out of plasma in situations of increased VP [4].

Although much effort has been devoted to developing drugs to directly block viral replication in VHF, countermeasures to address increases in VP have not been evaluated. With the exception of dengue virus infection in mice deficient in interferon response pathways [5,6], animal models to evaluate novel therapeutics to reverse endothelial vascular leakage have not been established. To develop VHF models of vascular leakage, it is necessary to establish methods to measure the process in living animals during the course of infection, as a means to evaluate the effects of agents employed to reduce the permeability of the endothelial cell lining.

For nearly a century, the intravenous infusion of Evans blue dye (EBD) has been a highly useful tool for evaluating blood volume [7,8]. Recent studies have employed EBD to measure vascular leakage in diseases of viral and non-viral origin in mice [5,6,9]. EBD binds to serum albumin with a high affinity, and thus leakage of plasma into the extravascular space can be determined during necropsy by removing organs and extracting the dye. The major limitation of this test is that, because the animals must be sacrificed, it can only be performed once during the course of illness. A non-invasive test for increased VP that could be used in conjunction with experimental therapies would be ideal to gauge the effects of a specific treatment.

An important consideration for modeling VHF is the need for a well-characterized system in which to evaluate the relationship between infection, viremia, the elaboration of proinflammatory mediators, increased VP, changes in intravascular volume and death. Although investigators have described a number of small animal models that reproduce various aspects of human VHF [10], none have examined vascular leakage in the context of the sequential events that lead to the death of the animals. Here we use a modified EBD procedure to detect increased VP and assess its connection with disease severity in a hamster model of arenaviral HF. We also validate the assay by demonstrating vascular leakage in hamsters infected with yellow fever virus. Having determined that the EBD assay can reveal increased VP during the course of acute arenaviral infection, we then assess whether changes in HCT and albumin, which can be measured in live animals, correlate with the timing of increased VP as shown by the EBD method. We also demonstrate for the first time, at the protein level, the hypercytokinemia induced by arenavirus infection in a hamster HF model.

## Methods

### Animals

Female golden Syrian hamsters were obtained from Charles River Laboratories (Wilmington, MA) and acclimated to the Laboratory Animal Research Facility at Utah State University for 3-6 days prior to use. Animals were ~6-8 weeks of age at time of challenge. All animal procedures complied with USDA guidelines and were approved by the Utah State University Institutional Animal Care and Use Committee.

### Viruses

Pichinde virus (PICV), strain An 4763, was provided by Dr. David Gangemi (Clemson University, Clemson, SC). The hamster-adapted Jimenez strain of yellow fever virus (YFV) was obtained from Dr. Robert Tesh (World Reference Center for Emerging Viruses and Arboviruses, University of Texas Medical Branch, Galveston, TX). The viruses were passed once through hamsters and stocks made from pooled hamster liver homogenates. Stocks were diluted in minimal essential medium (MEM, Hyclone, Logan, UT) just prior to infectious challenge (0.2 ml) by the intraperitoneal (i.p.) route.

### Assessment of vascular leakage

VP was determined by intravenous administration of EBD (Sigma-Aldrich, St. Louis, MO) and tracking its diffusion into various tissues. At designated times following infection, hamsters were anesthetized with isoflurane and injected retro-orbitally with a 0.5% EBD PBS solution. Retro-orbital injections were given using a 27-gauge needle by carefully inserting the tip ~1 mm from the outermost edge of the eye into the membrane exposed by gently pulling the skin away from the eye. Once the membrane has been penetrated, the solution is slowly injected to allow for rapid absorption by the capillary nexus at that site. The retro-orbital intravenous injection method has been compared to tail vein delivery in mice and found to be equally effective [11]. The amount of EBD injected was based on body weight with animals receiving ~18 mg/kg of dye (0.4 ml for a 110 g hamster). After a 4 h period, blood was collected in sacrificed animals by cardiac puncture, and the vasculature was extensively perfused transcardially with PBS to remove residual blood. Tissue sections of liver, spleen, lung, kidney and small intestine were harvested, weighed, and immersed in 0.5 ml of formamide for EBD extraction from tissue samples by overnight incubation at 37°C. A 0.1 ml volume from each sample tube was placed into a 96-well microtiter plate, and the absorbance at 610 nm was measured. Relative EBD content in the serum was determined from 1:10 diluted samples measured at 610 nm and 740 nm. The absorbance at 740 nm was subtracted from the 610 nm absorbance values to deduct the contributions from hemoglobin contamination. Data are expressed as a ratio of absorbance per g of tissue:absorbance of the diluted respective serum sample.

### Hematocrit (HCT), white blood cell (WBC), and other hematologic parameter analysis

Anticoagulated whole blood samples taken from hamsters were evaluated on a VetScan^® ^HMT analyzer (Abaxis, Union City, CA) to assess HCT, WBC, and other parameters. At designated times following infection, groups of hamsters were anesthetized with isoflurane for retro orbital venous sinus blood collection (~0.5 ml) or sacrificed for blood draw by cardiac puncture. Only HCT was evaluated in the YFV system. PICV studies included the analysis of WBC, red blood cells (RBC), and hemoglobin (Hb) from repeated blood collection during the infection.

### Blood chemistry

Sera collected on day 7 of PICV infection from two independent experiments (n = 10) were stored at -80°C until time of analysis. A VetScan VS2 analyzer (Abaxis, Inc., Union City, CA) was used to determine blood chemistry values. The Comprehensive Diagnostic Profile reagent rotor was selected for quantitative determination of 14 parameters (ALB, ALP, ALT, AMY, BUN, CA^++^, CRE, GLOB, GLU, K^+^, NA^+^, PHOS, TBIL, TP) described in the footnotes of Table 1. For comparison, serum samples from 6 sham-infected hamsters from the same two experiments were also analyzed.

### Serum albumin measurement

Sera obtained from a time course study of PICV infection in hamsters were analyzed for albumin content using the Albumin Reagent Set from Pointe Scientific (Canton, MI) per the manufacturer's recommendations. The assay was adapted for use in 96-well microtiter plates by adjusting the reagent volumes. Albumin standard was also obtained from Pointe Scientific.

### Multi-cytokine, chemokine, and serum factor analysis

Serum samples from PICV-infected and sham-infected groups of hamsters were sent to Rules-Based Medicine, Inc. (RBM, Austin, Texas) for analysis of 58 biomarkers on the RodentMAP^® ^version 2.0 platform (Additional file 1, Figure S1), which employs microspheres impregnated with fluorescent dyes and coated with reagents that bind with target substances in serum and plasma. Using this technology, RBM can analyze a large number of serum factors in less than 50 μL of serum. Although the RodentMAP^® ^is only validated for mouse samples, we have previously observed that antibody cross-reactivity enables the system to measure relative levels of a number of hamster proteins (B. Gowen, unpublished data). Nevertheless, the absence of measurable changes in certain cytokines or factors in our analysis of hamster samples may be due to limited cross-reactivity, and not necessarily a lack of involvement. The least detectable dose (LDD) is defined as 3 standard deviations above mean background measured for each analyte in each multiplex. If an analyte's value is below the LDD but still falls on the calibration curve, the value is reported. These values are likely real, but the precision of these values are decreased when they fall below the LDD.

## Results

### Measurement of increased VP in PICV-infected hamsters

**Figure 1:**
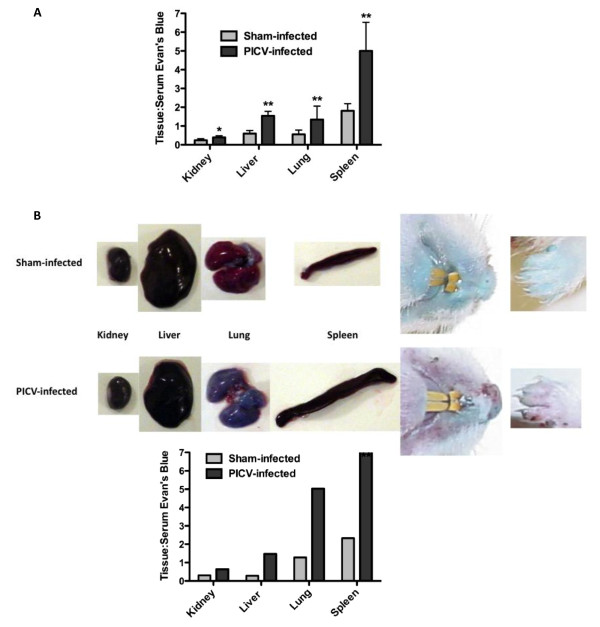
**Evidence of increased vascular permeability during late-stage PICV infection in hamsters**. A) Two groups of animals (n = 6/group) were infected with ~5 plaque-forming units of PICV or sham (MEM vehicle). On day 7 of infection, EBD-PBS was injected retro-orbitally and the ratio of EBD concentration in the kidney, liver, lung and spleen to the serum EBD concentration was evaluated. Data are expressed as the ratio of absorbance/g of tissue:absorbance of the diluted matching serum sample. *P < 0.05, **P < 0.01 compared to sham-infected hamsters by the Mann-Whitney test (two-tailed). B) Images taken of kidneys, livers (single lobes), lungs, and spleens harvested from a sham-infected control and a PICV-infected hamster on day 7. The serum concentration of EBD was 34% greater in the sham-infected control compared to the infected animal. On the right, images of the respective hamsters' mouths and front paws are included to show the stronger blue coloration notable in the uninfected animal. Dye was extracted from the kidneys, livers, lungs, and spleens shown and the EBD content data are represented in the associated graph.

Our initial goal was to test the hypothesis that increased VP is associated with severe PICV infections in hamsters. To this end, we modified a previously described method used in mice based on the tracking of EBD [5] injected i.v. through the tail vein to measure systemic leakage into various tissues. First, hamsters had to be infused by the retro-orbital route. To control the animal-to-animal variation that may be associated with EBD-PBS injected retro-orbitally, we modified the procedure to normalize our measurements of tissue EBD concentrations by dividing each tissue value by the serum EBD concentration. As shown in Figure 1A, a significant increase in the tissue/serum EBD ratio for liver, spleen, lung, and kidney were observed in PICV-infected animals on day 7, relative to sham-infected controls. A dramatic difference in blue coloration is evident upon gross examination of the internal organs, with the lungs of PICV-infected animals showing a striking blue coloration, compared to those of healthy controls (Figure 1B). Moreover, the data obtained from the EBD extractions from the tissues shown in Figure 1B support the visual evidence that increased VP is associated with advanced stage PICV infection in hamsters, while the EBD serum content was typically higher in uninfected than in PICV-infected hamsters. For example, in the animal shown in Figure 1B, the serum EBD concentration was 34% less in the infected animal than in the sham-infected control, consistent with the leakage of dye into the viscera. Remarkably, in contrast to infected animals, the skin of healthy hamsters turned blue within several hours of EBD injection, consistent with the retention of the dye within the vascular system.

### Time course analysis of VP during PICV infection

**Figure 2:**
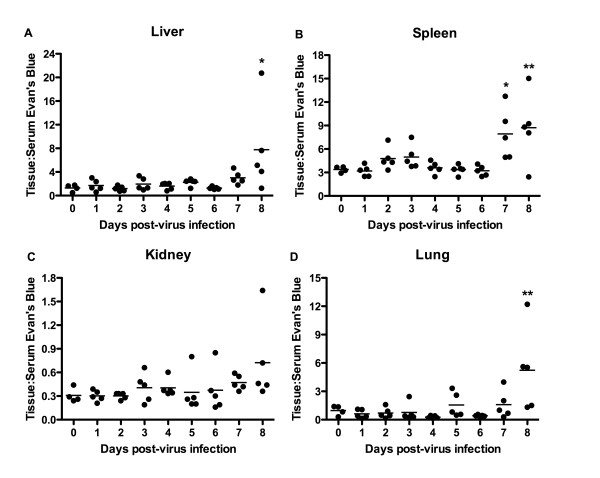
**Analysis of vascular permeability during PICV infection in hamsters**. Groups of animals were infected with ~5 plaque-forming units of PICV, with the exception of 4 hamsters that were processed at the time of infection to establish the day 0 baseline reading. On each day of the infection (days 1-8), a group of animals was injected retro-orbitally with EBD-PBS and vascular leak into the (A) liver, (B) spleen, (C) kidney, and (D) lung tissues was evaluated. Data are expressed as the ratio of absorbance/g of tissue:absorbance of the diluted matching serum sample. For days 1-6, there were 5 hamsters/group. Days 7 and 8 had 6 animals/group, but due to the death of one animal in each group prior to time of sacrifice, the analysis is based on 5 hamsters for these groups. *P < 0.05, **P < 0.01 compared to day 0 animals using one-way analysis of variance (ANOVA) with the Newman-Keuls multiple comparison test.

Studies aimed at resolving the timeframe in which increased VP occurs in the hamster PICV infection model are essential to inform future studies in which the initiation of treatment may be triggered by the onset of vascular leakage. To this end, we conducted an experiment to gauge vascular leakage during the acute infection in hamsters. A statistically significant increase in the ratio of tissue:serum EBD concentration was seen on day 7 following PICV challenge in the spleen and on day 8 in the liver, spleen, and lungs (Figure 2). Notably, the animal that had lost the most weight in the day-8 sacrifice group (data not shown) had the highest levels of EBD in liver, kidney and lung, but not spleen tissue.

Based on the previous experiment, (Figure 1A), we had expected to see a greater increase in vascular leak on day 7 in liver and lung. However, it is important to note that one animal in each of the last two sacrifice groups (day 7 and 8) succumbed to the infection after EBD-PBS administration (day 7 hamster) or prior to the day of sacrifice (day 8 hamster). Because the data presented in Figure 2 lack samples from very ill animals, they probably underestimate the vascular leakage for those groups. This fact, and experimental variability comparing samples harvested on different days, could account for the less pronounced increase in VP that we observed in this study.

### Longitudinal analysis of WBC, HCT, and other hematologic parameters during PICV infection

**Figure 3:**
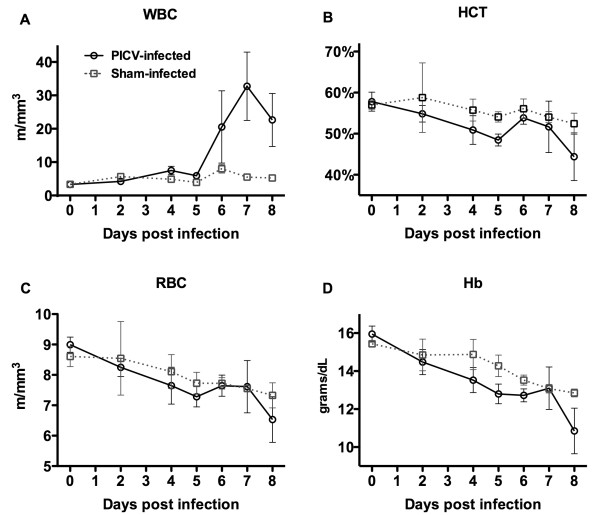
**Longitudinal hematologic analysis of PICV infection in hamsters**. Two groups of hamsters were infected with ~5 plaque-forming units of PICV or sham-infected. On each of days 0, 2, and 4-8 of the infection, blood was collected by retro-orbital venous sinus route following isoflurane anesthesia for hematologic analysis as described in the methods section. The data points represent the analysis of samples from groups of 4 hamsters with the exception of the PICV-infected group, wherein due to the death of a single animal in the on day 7, and a second hamster on day 8, the data reflect values for 3 and 2 animals, respectively, for days 7 and 8. (A) White blood cells, WBC, (B) hematocrit, HCT, (C) red blood cells, RBC, and (D) hemoglobin, Hb values are shown. m/mm^3^, millions per cubic milliliter of blood.

Increased circulating levels of WBC, reflecting their mobilization from the bone marrow, are a reliable indicator of viral infection. We previously showed that in PICV-infected hamsters, virus becomes detectable in the circulation on day 5 of infection [12]. Consistent with this finding, we observed an increase in the WBC count starting on day 6 and remaining high through day 8 (Figure 3A).

We also tracked changes in HCT in PICV-infected hamsters. We anticipated seeing an increase in HCT, reasoning that as vascular leakage occurs there would be an increase in the packed cell volume due to plasma volume loss, but probably because daily samples were collected from individual animals on days 0, 2, and 4-8, the HCT actually dropped slightly over the course of the infection (Figure 3B). Because one infected animal died on day 7 and one on day 8, the mean HCT values on those days may be underestimated.

The main objective of this experimental approach was to determine whether the HCT could serve as a marker for VP over the course of illness. Because frequent blood collection led to anemia, as determined by the RBC count and hemoglobin content (Figure 3C, D), we also conducted a study in which we collected blood samples only on days 6 and 7 of infection, which coincides with the transition to loss of vascular integrity by the EBD method. In the day 6 samples (3 sham- and 5 PICV-infected), the HCT was significantly higher in the sham-infected animals (data not shown), similar to the trend seen in the earlier blood sampling study (Figure 3B). On day 7, there was no significant difference between the sham- and PICV-infected hamsters (data not shown). It is possible that RBC loss from the intravascular compartment in infected animals may be occurring through extravasation into tissues. We investigated this possibility by histological analysis of liver, lung, and spleen sections harvested on day 7 of infection but did not see evidence of RBC loss by this mechanism (data not shown). Our findings, and that previously reported for Pirital arenavirus infection in hamsters [13], indicate that changes in the HCT do not directly reflect alterations in VP.

### Blood chemistry analysis and reduced serum albumin as a marker for vascular leakage

Because the binding of EBD to albumin is the basis of the test for vascular leak, we reasoned that changes in the serum albumin concentration might be used to track alterations in VP. We therefore performed a blood chemistry analysis to assess its utility in hamsters. As shown in Table 1, the serum albumin level was significantly reduced in PICV-infected hamsters on day 7, compared to the sham-infected controls.

**Table 1:** Mean blood chemistry values from sham- or PICV-challenged hamsters on day 7 of infection^a^.

Analyte	Sham-infected	PICV-infected	*P*-value^b^
**ALB (g/dL)**	**5.2 ± 0.2**	**4.0 ± 0.3**	***
**ALP (U/L)^c^**	**180 ± 27**	**> 1914 ± 606**	***
**ALT (U/L)^d^**	**160 ± 74**	**> 1894 ± 213**	***
**AMY (U/L)**	**1659 ± 408**	**690 ± 227**	***
TBIL (mg/dL)	0.23 ± 0.05	0.62 ± 0.61	NS
BUN (mg/dL)	25.8 ± 1.7	24.2 ± 13.4	NS
**Ca^++ ^(mg/dL)**	**14.2 ± 0.6**	**13.2 ± 0.8**	*
**PHOS (mg/dL)**	**10.6 ± 0.6**	**8.2 ± 1.0**	***
**CRE (mg/dL)^e^**	**< 0.25 ± 0.05**	**< 0.38 ± 0.12**	*
**GLU (mg/dL)**	**183 ± 30**	**80 ± 37**	***
Na^+ ^(mM)	148 ± 4	149 ± 4	NS
K^+ ^(mM)^f^	> 8.5 ± 0.0	> 8.4 ± 0.1	NS
TP (g/dL)	6.3 ± 0.1	6.3 ± 0.4	NS
**GLOB (g/dL)**	**1.1 ± 0.3**	**2.3 ± 0.5**	***

^a^Sham-infected, n = 6; PICV-infected, n = 10.

^b^Student's two-tailed t-test. *P < 0.05, **P < 0.01, ***P < 0.001; NS, not significant.

^c^Three PICV-infected hamsters had saturated levels of ALP (> 2400 U/L).

^d^Seven PICV-infected hamsters had saturated levels of ALT (> 2400 U/L).

^e^One PICV- and three sham-infected hamsters had levels below the limit of detection (0.2 mg/dL)

^f^Seven PICV- and six sham-infected hamsters had saturated levels of K^+ ^(8.5 mM).

**Bold **font indicates analytes differing significantly between sham- and PICV-infected hamsters.

ALB, albumin; ALP, alkaline phosphatase; ALT, alanine aminotransferase; AMY, amylase; TBIL, total bilirubin; BUN, blood urea nitrogen; Ca^++^, calcium; PHOS, phosphate; CRE, creatinine; GLU, glucose; Na^+^, sodium; K^+^, potassium; TP, total protein; GLOB, globulin.

**Figure 4:**
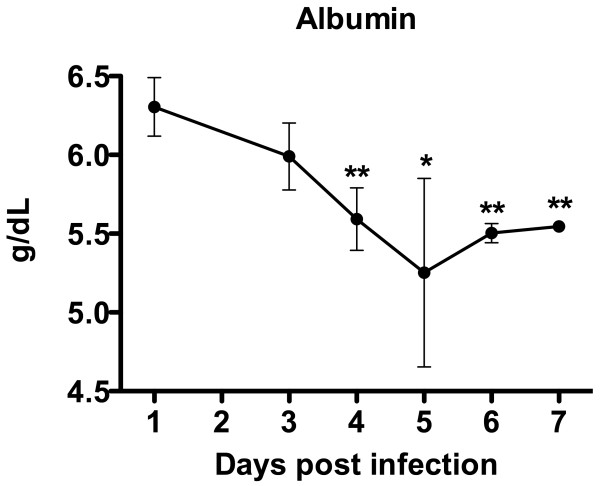
**Serum albumin levels during the course of PICV infection in hamsters**. Groups of hamsters were infected with ~5 plaque-forming units of PICV and serum was obtained following sacrifice on days 1, and 3-7 of PICV infection. Samples were assayed for albumin concentration. Data represent the means and standard deviations of 3-4 hamsters per group. *P < 0.05, **P < 0.01 compared to day 0 animals using ANOVA with the Newman-Keuls multiple comparison test.

Previously, evidence of decreased albumin levels in VHF hamster models based on infection with the related Pirital arenavirus [13] and YFV [14,15] has been reported. Having observed dramatically reduced serum albumin levels in the PICV-infected animals, we further evaluated the possibility of using this analyte as a non-terminal indicator of vascular perturbation. A more rapid and cost-effective assay was employed to specifically evaluate serum albumin concentration during the course of PICV infection. As observed in Figure 4, serum albumin levels drop significantly by day 4 and remained low through the end of the 7-day sampling period. Although there are differences in the g/dL amounts estimated by the two albumin detection methods (VetScan VS2 analyzer and Pointe Scientific Albumin Reagent Set), clear differences relative to the respective controls were observed.

Blood chemistry analysis also revealed a number of other changes during PICV infection (Table 1). As expected, concentrations of the liver-associated enzymes ALP and ALT were extremely high in the infected animals. Amylase was markedly decreased, as were calcium, phosphate, and glucose levels. It is conceivable that the decrease in glucose levels is due to a reduction in food consumption as the animals become sick and perhaps because the liver and pancreas are diseased and malfunctioning. Pancreatic insufficiency would explain the lower amylase concentrations. Also as expected, globulin levels were significantly elevated on day 7, which is generally one day before the hamsters begin to succumb.

### Profiling of hamster serum cytokines, chemokines and other serum factors during PICV infection

**Figure 5:**
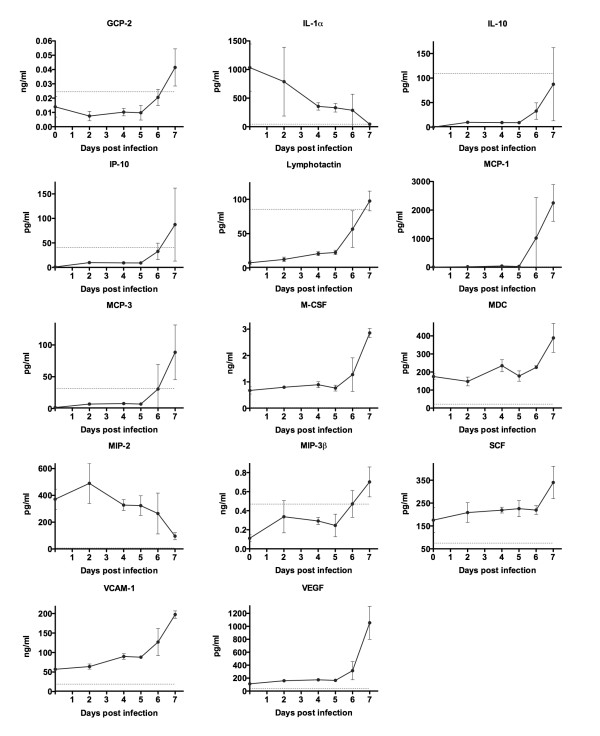
**Analysis of systemic levels of inflammatory mediators during PICV infection in hamsters**. Groups of animals (n = 3-4/group) were infected with ~5 plaque-forming units of PICV, with the exception of 3 hamsters that were processed at the time of infection to establish the day 0 baseline reading. On each of days 2, and 4-7 of the infection, serum was collected from PICV-infected hamsters for multi-analyte profiling of serum antigens. Data are shown for hamster cytokines and chemokines that were sufficiently cross-reactive with the Rodent MAP^® ^detection platform and changed significantly over the course of infection. The least detectable dose is indicated by the red hashed line and is defined in the methods. GCP, Granulocyte Chemotactic Protein; IL, Interleukin; IP, Inducible Protein; MCP, Monocyte Chemoattractant Protein; M-CSF, Macrophage-Colony Stimulating Factor; MDC, Macrophage-Derived Chemokine; MIP, Macrophage Inflammatory Protein; SCF, Stem Cell Factor; VCAM, Vascular Cell Adhesion Molecule; VEGF, Vascular Endothelial Cell Growth Factor.

In severe viral infections, hypercytokinemia triggers the changes in VP that lead to leakage of plasma into the extravascular spaces and circulatory collapse. In the present study and elsewhere [12], we have demonstrated the sequence of viremia beginning on day 5 of PICV infection, followed by the appearance of elevated WBC on day 6, and transition to vascular leakage on day 7 and 8. In order to determine whether proinflammatory mediators could play a causative role in this process, we analyzed serum from sham- and PICV-infected hamsters on days 0, 2, and 4-7. As shown in Figure 5, there was a substantial increase from day 5 to 6 in many of the cytokines and chemokines examined, preceding the increase in VP measure by the EBD method (Figure 2). Interestingly, IL-1α and MIP-2 showed a reversal in the predominant trend, as these factors decreased through the course of infection.

Of the non-cytokine/chemokine factors evaluated, three were altered during the infection (Additional file 2, Figure S2). Fibrinogen, an acute phase protein, was markedly elevated from baseline values of ~200 μg/ml through the first 5 days of infection, up to ~30,000 to 70,000 μg/ml on days 6 and 7. Consistent with previously reported data [12], aspartate aminotransferase (AST) concentration, a prognostic indicator of disease outcome in human cases of Lassa fever [16], increased gradually over time with a spike on day 6. Also dramatically increased beginning on day 6 after infection was Von Willebrand factor (vWF).

### Analysis of vascular leakage in hamsters infected with YFV

**Figure 6:**
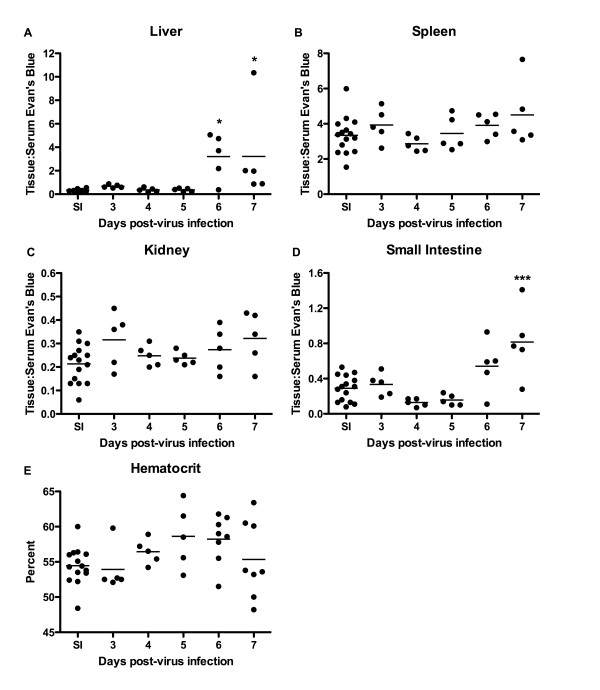
**Analysis of vascular permeability and HCT during YFV infection in hamsters**. Groups of animals were infected with ~20 cell culture infectious doses (CCID_50_) of YFV or sham-infected. On specified days of the infection (days 3-7), groups of YFV-infected hamsters (n = 5) and groups of sham-infected animals (n = 3) were injected retro-orbitally with EBD following collection of whole blood for evaluation of HCT. Vascular leak into the (A) liver, (B) spleen, (C) kidney, and (D) small intestine tissues, and (E) HCT were evaluated. Vascular permeability data are expressed as the ratio of absorbance/g of tissue:absorbance of the diluted matching serum sample. SI, sham-infected. *P < 0.05, ***P < 0.001 compared to the pool of sham-infected animals using ANOVA with the Newman-Keuls multiple comparison test.

To investigate whether another established hamster model of VHF would show evidence of increased VP measured by the transfer of EBD from serum to the internal organs, YFV-infected hamsters were evaluated from 3-7 days post infection to assess the possible development of vascular leak. A significant increase in the ratio of tissue to serum EBD concentration was seen in the liver and small intestine of infected animals, beginning on day 6 and continuing through day 7 (Figure 6A, D). Clear indication of EBD leakage into spleen and kidney tissue was not observed (Figure 3B, C). Previously, slightly increased HCT levels on day 5 of YFV infection in hamsters have been reported [17,18]. In the present study, the HCT similarly increased slightly on days 5 and 6, but the difference compared to sham-infected controls was not statistically significant (Figure 3E). No change in HCT was seen on day 7, when the most prominent change in EBD concentration in the liver and small intestine of infected animals was observed, suggesting that factors other than VP are affecting the HCT.

## Discussion

This report marks the first attempt to assess changes in vascular barrier function during acute infections in hamsters that model VHF. Vascular barrier function is controlled through inter-endothelial cell contacts made, for example, of adherens junctions such as vascular endothelial cadherin (VE-cadherin) [19]. Under normal conditions, these junction proteins adequately regulate fluid leak through strong homophilic contacts. However, during pathologic settings such as VHF, when the vascular barrier is responding to exaggerated and sustained signals promoting increased VP, the result is excessive edema accumulation, hypotension, and ultimately death. As is the case for other diseases such as sepsis and pandemic influenza, VHF influences vascular barrier function through increased levels of circulating cytokines. These mediators negate VE-cadherin function, leading to vascular hyperpermeability, multiorgan edema and failure, non-cardiogenic shock and death. Here, we use the transfer of EBD from the plasma into the viscera as a marker to demonstrate that viremia leads to increased cytokine levels, followed by enhanced VP, in the PICV hamster model of arenaviral HF. Significant accumulation of EBD indicative of vascular hyperpermeability in both the PICV and YFV models precedes mortality, suggesting that platforms that enhance vascular stability may be an excellent therapeutic system to target late stage events seen during disease progression.

We evaluated different methods of detecting changes in VP in the PICV hamster infection model. Further, we report the first profiling of systemic protein levels of a large number of cytokines, chemokines, and other serum factors in the PICV hamster model. The data provide insights into the contribution of the excessive proinflammatory response that induces vascular hyperpermeability associated with VHF and sepsis. This type of understanding supports the use of this model system to study antiviral therapies and pathophysiology, and it brings to focus issues associated with drug delivery during advanced disease wherein compromised drug absorption profiles may be observed when dosing by routes of administration commonly used in animal experiments (i.p., i.m., and s.c.). Challenges still lie ahead in regards to the evaluation of therapeutic approaches aimed at preventing increases in VP in the face of a severe viral infection. The major hurdle is the lack of a good marker of vascular hyperpermeability that can be measured in live animals to assess the impact of a candidate therapy on correcting the problem.

The present study describes our efforts to identify markers of increased VP in a hamster model of acute arenaviral infection. Our findings do not support the use of the HCT as a measure of vascular leakage in the hamster PICV infection models. Because we did not monitor water consumption of individual hamsters, it is possible that the animals continued to drink sufficient amounts of water, thereby limiting the elevation in HCT at the later stages of infection. However, we don't believe that this occurred, because the animals began to lose weight on day 7, suggesting that water and food intake was greatly reduced. In addition, there was no evidence of RBC loss due to extravasation into viscera of infected hamsters. There may also be some intravascular hemolysis occurring as the serum total bilirubin levels are increased, but not statistically significantly. This increase in total bilirubin has also been noted during Pirital arenavirus infection in hamsters [13]. It is also possible that alterations in normal kidney function due to infection may reduce the production of erythropoietin, thereby curtailing RBC production and masking hemoconcentration. Collectively, the potential contributions of multiple factors do not make HCT a good marker for measuring VP in PICV-infected hamsters.

A low serum albumin concentration was also considered as a marker for increased VP. However, the fact that albumin levels decreased significantly by day 4 following PICV challenge, while serum cytokine levels were still normal, indicates that, as in the case of HCT, the serum albumin concentration is influenced by factors other than VP. By the current "gold standard" of measuring EBD leakage, we do not see evidence of vascular perturbation until day 7 and 8 of PICV infection. The apparent dissociation with drop in albumin and changes in VP may be due to decreased hepatic synthesis of albumin as a result of altered liver function in PICV-infected animals. This possibility is supported by previously published data showing 5 log_10 _of liver virus burden starting on day 2 through 4 of infection, with 6 log_10 _of virus by day 5, and 8 log_10 _by day 6 [12]. Notably, with the YFV model, serum albumin drops significantly on days 5 and 6 of infection [14,15], which is more proximal to the time at which we observed evidence of vascular leak (day 6 in the liver and day 7 in the small intestine) by the EBD method.

Measurement of the tissue/serum EBD ratio would appear to be the best method of assessing the effect of an experimental therapy aimed at countering increased VP. Our normalization of the data through the conversion of EBD concentration into a tissue/serum ratio controls for animal-to-animal variability in dosing, and provides a more meaningful determination of the transfer of dye from the serum into the organs than the tissue concentration alone. At the same time, the retention of EBD within the vascular system of healthy animals, causing their skin to develop a strong blue color, serves as a readily visible marker of normal vascular function. The degree of blue coloration of the skin could thus be scored semi-quantitatively on a scale of 0 to 4 at a fixed time after EBD infusion, with a low score indicating the development of increased VP. Because the dye is not toxic at the doses used and is cleared very slowly from the circulation, animals could be monitored continually to evaluate vascular function. However, one must consider any possible effects of EBD on the model system, drug-dye interactions, and the fact that in a few cases, EBD has been shown to have antiviral activity *in vitro *[20-22].

**Figure 7:**
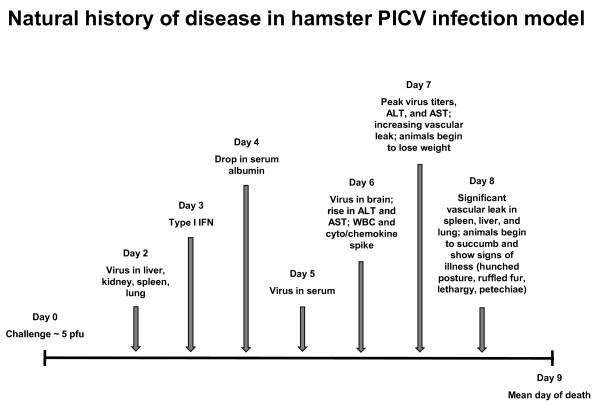
**Natural history of disease in hamster PICV infection model**. The schematic shown is a summary of the present data integrated with previously published findings [12]. Two days after challenge, the presence of PICV in the liver, kidney, spleen, and lung is evident. On day 3, the type I IFN response is measurable and fluctuates slightly over the duration of the acute infection. On days 4 and 5, a significant drop in serum albumin and the first signs of systemic PICV burden, respectively, are observed. By day 6, infectious PICV can be detected in the brain, ALT and AST enzyme levels rise, and WBC and proinflammatory mediators dramatically increase. Day 7 marks the onset of vascular leak, peak viral titer, ALT, and AST concentrations, and initial signs of weight loss. Eight days after challenge, significant vascular leakage is present in multiple tissues, with some animals beginning to succumb and showing clear signs of illness. The majority of the hamsters will succumb by day 9 of infection.

Our studies of PICV and YFV infection of hamsters suggest that these models reproduce the vascular leakage that plays a key role on the pathogenesis of VHF leading to vascular collapse [1,23]. In the PICV hamster infection model, the basic features of VHF in humans are demonstrated through the sequence of viremia, followed by excessive release of inflammatory mediators, vascular hyperpermeability and death (Figure 7). These models therefore offer an opportunity to propose and test new therapeutic strategies, including those that prevent or correct the increase in VP that is associated with severe disease and death in VHF [2,24]. Strategies aimed at reducing viremia or immunomodulation continue to dominate. As other models of VHF are characterized with a focus on the timing and pathologic events between infection and death, we may find that an approach based on blunting the effects of excessive inflammatory mediators on vascular function may provide the basis for a broad based strategy to treat multiple infectious challenges [19,25].

## Competing interests

The authors declare that they have no competing interests.

## Authors' contributions

All authors read and approved the final manuscript. BBG, JGJ, and MB conceived and designed the experiments. MHW and DL performed the experiments. BBG, JGJ, NRL, JDM, DYL and MB analyzed the data. BBG and MB wrote the paper.

## Supplementary Material

Additional file 1 **Figure S1: Rodent MAP^® ^antigens**. Complete 58 antigen panel of the version 2.0 Rodent MAP^® ^system validated for interrogation of mouse serum or plasma samples. Sufficient cross reactivity of hamster factors allows for detection and measurement of relative levels for many of the antigens. Click here for file

Additional file 2 **Figure S2: Changes in systemic levels of fibrinogen, AST, and vWF during PICV infection in hamsters**. Groups of animals (n = 3-4/group) were infected with ~5 plaque-forming units of PICV, with the exception of 3 hamsters that were processed at the time of infection to establish the day 0 baseline reading. On each of days 2, and 4-7 of the infection, serum was collected from PICV-infected hamsters for multi-analyte profiling of serum antigens. Data are shown for non-cytokine/chemokine factors that were sufficiently cross-reactive with the Rodent MAP^® ^detection platform and changed significantly over the course of infection. The least detectable dose is indicated by the red hashed line and is defined in the methods. AST, aspartate aminotransferase; vWF, Von Willebrand factor. Click here for file
