# Supplementary figures and images for: Assessing changes in vascular permeability in a hamster model of viral hemorrhagic fever
Source: Virol J. 2010 Sep 16;7:240. doi: 10.1186/1743-422X-7-240 (PMC2949842; doi:10.1186/1743-422X-7-240)

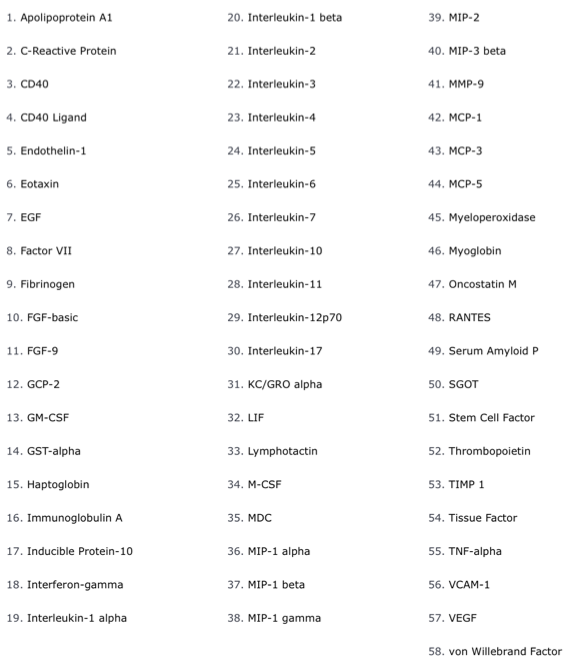

Supplement: Additional file 1 — Figure S1: Rodent MAP® antigens. Complete 58 antigen panel of the version 2.0 Rodent MAP® system validated for interrogation of mouse serum or plasma samples. Sufficient cross reactivity of hamster factors allows for detection and measurement of relative levels for many of the antigens. [file 1743-422X-7-240-S1.TIFF]

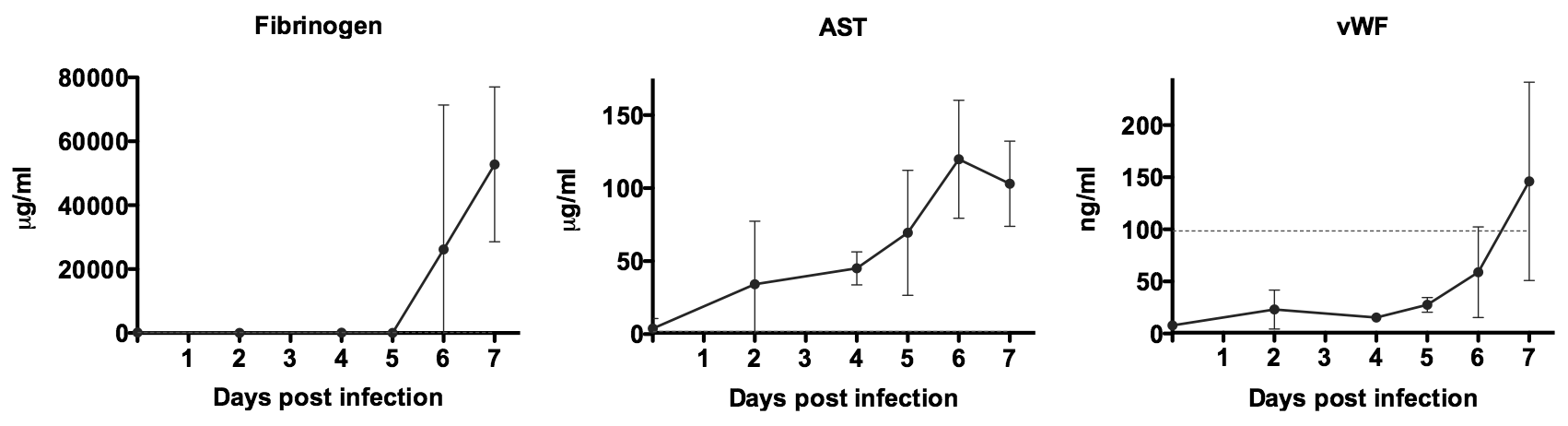

Supplement: Additional file 2 — Figure S2: Changes in systemic levels of fibrinogen, AST, and vWF during PICV infection in hamsters. Groups of animals (n = 3-4/group) were infected with ~5 plaque-forming units of PICV, with the exception of 3 hamsters that were processed at the time of infection to establish the day 0 baseline reading. On each of days 2, and 4-7 of the infection, serum was collected from PICV-infected hamsters for multi-analyte profiling of serum antigens. Data are shown for non-cytokine/chemokine factors that were sufficiently cross-reactive with the Rodent MAP® detection platform and changed significantly over the course of infection. The least detectable dose is indicated by the red hashed line and is defined in the methods. AST, aspartate aminotransferase; vWF, Von Willebrand factor. [file 1743-422X-7-240-S2.TIFF]
